# Leapfrog migration and residents: New migratory habits in Swedish Greylag geese

**DOI:** 10.1002/ece3.8740

**Published:** 2022-03-23

**Authors:** Lovisa Nilsson, Camilla Olsson, Johan Elmberg, Nils Bunnefeld, Niklas Liljebäck, Johan Månsson

**Affiliations:** ^1^ Grimsö Wildlife Research Station, Department of Ecology Swedish University of Agricultural Sciences Riddarhyttan Sweden; ^2^ Department of Environmental Science and Bioscience Kristianstad University Kristianstad Sweden; ^3^ Biological and Environmental Sciences University of Stirling Stirling UK

**Keywords:** animal movement, *Anser anser*, flyway management, GPS telemetry, individual variation, net squared displacement

## Abstract

Knowledge about intraspecific and individual variation in bird migration behavior is important to predict spatiotemporal distribution, patterns of phenology, breeding success, and interactions with the surrounding environment (e.g., human livelihoods). Such variation is key to adaptive, evolutionary responses, i.e., how individuals respond spatiotemporally to the environment to maximize fitness. In this study we used GPS location data from one to three full annual cycles from 76 Greylag geese (*Anser anser*) to test the hypothesis that geese originating at five latitudinally separated capture sites in Sweden have different migration strategies. We also assessed individual consistency in movement strategy over consecutive annual cycles. We used the scale‐independent net squared displacement modeling framework to quantify variables of autumn and spring migration for geese from each capture site: distance, timing, and duration. Our results demonstrate a positive correlation between migration distance and latitudinal origin. Geese from the northernmost site on average migrated farther south and about 15 times as far as the short‐moving or resident geese from the two southernmost sites. Movement strategies of individual geese varied considerably both within and among capture sites. Individual consistency in movement strategy from one annual cycle to the consecutive was high in geese from the northern sites moving the farthest, whereas the resident or short‐moving geese from the southernmost sites generally showed lower or no individual consistency. These changes have come about during a time span so short (i.e., ca. 35 years or 8–10 generations) that it can unlikely be explained by classical Darwinian between‐generation adaptation. Consequently, and given that young geese follow their parents during their first migration, we presume an important role of within‐family, inter‐generation change as a driver behind the large‐scale changed migration habits in Swedish Greylag geese.

## Graphical abstract

Knowledge about intraspecific and individual variation in bird migration behavior is key to predict adaptive and evolutionary responses, i.e., how individuals respond spatiotemporally to the environment to maximize fitness. In this study we used GPS location data from one to three full annual cycles from 76 Greylag geese, *Anser anser*, to test the hypothesis that geese originating at five latitudinally separated capture sites in Sweden have different migration strategies. Our results demonstrate that migration strategies of individual geese varied considerably both within and among capture sites, with increasingly longer migration distances for geese originating at higher latitudes.

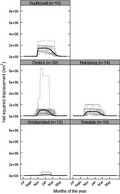

## 1 INTRODUCTION

Knowledge about intraspecific individual variation in migration behavior is vital to understand patterns of phenology, changes in breeding success, resource use, and interactions with the environment (e.g., habitats, other species, human livelihoods). Such variation is also key to adaptive responses studied within the evolutionary paradigm; i.e., how individuals respond spatiotemporally to selective regimes in an optimal way to maximize fitness (Alerstam & Hedenström, 1998). Accordingly, there has been a gradual historical shift in the general view on animal migration, from stereotyped patterns to a deeper appreciation of intraspecific variation as a driver of migration dynamics in populations. For example, migration distance in species in which all individuals migrate may vary by sex, age, or body condition; a process and pattern termed “differential migration” (Gauthreaux, 1982; Ketterson & Nolan, 1983; Newton, 2008). Similarly, some species are “partial migrants,” where some individuals are true migrants, others do not migrate at all. In this case, migration strategy may differ by age, body condition, genetic constitution, or the frequency of a certain strategy in the population (Berthold, 2001; Newton, 2008).

Aside from short‐term events (challenging weather, food shortage, etc.) that force individuals to change distance or timing of migration, it may also change because of evolutionary adaptation to long‐term environmental change (e.g., land use, climate). Research accumulated over the last two decades documents significant changes in timing of migration in a wide range of avian species (Lehikoinen et al., 2019; Møller et al., 2010). This is mainly manifested by migrants arriving earlier than before to breeding grounds in spring, and in some species by a later departure in autumn (Jonzén et al., 2006; Lehikoinen et al., 2019; Mills, 2005). Migration distance per se has shrunk in several species, as witnessed by decreased mean recovery distance due to long‐term climate change in 12 of 24 bird species ringed in the Netherlands (Visser et al., 2009). A related and widespread phenomenon is a northward shift in wintering range in medium‐ and short‐distance migrants in temperate areas of the northern Hemisphere. This has been documented in passerines, shorebirds, raptors, and waterfowl (Pavón‐Jordán et al., 2019; Potvin et al., 2016), and exemplifies “winter partial short‐stopping” *sensu* Elmberg et al. (2014). However, less is known to what extent seasonal range shifts across a migratory flyway affect different populations or individuals within a population similarly. Previous research on differential and partial migration patterns as well as recent studies on range shifts are often based on crude arrival and departure dates or on census data. Consequently, they embrace little information about individual movements and intra‐population differences, i.e., the potential drivers of the shift. Although time‐series data collected over larger areas are crucial to infer changed migratory habits in the first place, such data inevitably contain noise and biases (Lehikoinen & Sparks, 2010). These deficiencies can be overcome by studying movements of individually tracked birds emanating from different source areas and followed throughout the annual cycle, an approach recently made possible for large‐ to medium‐sized birds such as geese by solar‐powered GPS technology.

Greylag geese breeding in Sweden were historically obligate long‐distance migrants, wintering in Coto Doñana in Spain and to a lesser extent in France (Follestad et al., 2001; Fransson & Pettersson, 2001). Analyses in the 1990s of banding recoveries demonstrated a classical “chain migration pattern” (i.e., “parallel migration” *sensu* Salomonsen, 1955) in which migration distance does not differ among birds breeding at different latitudes, so that northern breeders winter north of southern breeders (Follestad et al., 2001; Fransson & Pettersson, 2001). The latter reference also reported a slight decrease in the relative share of recoveries from southern Europe in winter in the late 1900s. Later studies reported winter partial short stopping from more southern populations in the same flyway of Greylag geese (Månsson et al., 2022; Podhrázský et al., 2017; Ramo et al., 2015). In addition, there has been a recent and rapid increase in Greylag geese wintering in southern Sweden, some of which are known local breeders (Nilsson & Kampe‐Persson, 2018a; Nilsson et al., 2020). Evidence from throughout the flyway thus indicates recent significant changes in migration habits in Greylag geese in Western Europe. These changes are often seen as an adaptive response to an increasingly benign environment with shorter and milder winters, reduced nutritional bottlenecks due to changed agricultural practices, and reduced per capita hunting mortality (Fox & Madsen, 2017; Fox et al., 2005). This provides opportunity to study adaptive changes in migration strategy in geese breeding at different latitudes within a flyway.

In this study, we provided Greylag geese caught on Swedish breeding grounds with neckbands equipped with solar‐powered GPS‐tracking devices. GPS location data from these birds not only confirmed a continued trend for winter short stopping but also showed that the change in migratory strategy differed depending on breeding latitude (Månsson et al., 2022). According to these data, northern breeders are still long‐distance migrants, whereas birds from the two southernmost capture sites show more limited winter movements (Månsson et al., 2022). These findings indicate that Greylag geese breeding in Sweden may have switched from a “chain migration” to a “leapfrog” pattern, in which northern breeders overshoot southern breeders in autumn and winter, and migration distance increases with breeding latitude (*c*.*f*., Salomonsen, 1955, Alerstam & Hedenström, 1998, Newton, 2008).

However, the mapped individual movement trajectories and the monthly mean locations for geese from different capture sites presented in Månsson et al. (2022) warrant evaluation by a more rigorous and objective analytical tool. The net squared displacement (NSD) statistical framework sets out to“(i) separate migration from other movement behaviours, (ii) quantify migration parameters without the need for arbitrary cut‐off criteria and (iii) test the predictability across individuals, time and space” (Bunnefeld et al., 2011). NSD has been used successfully to study drivers of migration in a wide range of taxa, mainly ungulates and carnivores, but also geese and cranes (Bunnefeld et al., 2011; Leopold & Hess, 2014; Smereka et al., 2021; Wolfson et al., 2020). For a Greylag goose population undergoing changes in migratory habits, NSD can be used to differentiate migrants from residents and to quantify key migration parameters (i.e., distance, timing, and duration). It can also be used to assess individual consistency in migratory habits between years.

In this study we used daily GPS location data from one to three full annual cycles obtained from 76 Greylag geese to test the hypothesis that birds originating at different latitudes have different migration strategies, so that the Swedish population have shifted from a chain migration to a leapfrog migration pattern. Based on the leapfrog hypothesis (Berthold, 2001; Salomonsen, 1955), we predicted that geese breeding the farthest north would winter the farthest south, whereas birds breeding the farthest south would move relatively shorter distances and winter the farthest north. The alternative hypothesis is that partial winter short stopping would affect all populations equally, leading to a retained pattern of chain migration. When it comes to individual consistency in migration strategy (i.e., distance, timing, and duration; Bunnefeld et al., 2011), we hypothesized that it would not change between years.

## 2 METHODS

### 2.1 Capture procedure and sites

The study is based on location data obtained 2017–2020 from 82 Greylag geese with GPS‐equipped neckbands, made by either Ornitela (OT‐N35 and OT‐N44) or Made‐by‐Theo (Theo Gerrits) (Table 1 and Table S3), placed in June (2017–2019), focusing on breeding and molting adults and their unfledged goslings. Birds were caught early in the morning when foraging in fields, pastures, or lawns near water. They were herded slowly by foot or canoe via fences into net corrals, where they were immediately put in gunny sacks to keep calm until further handling. Handling protocols were approved by the animal ethics committee for central Sweden and fulfilled the ethical requirements for research on wild animals (decision Dnr 5.8.18‐03584/2017).

**TABLE 1:** Number of Greylag geese (*n *= 76) with movement data (>349 locations per annual cycle) per capture site and annual cycle

Capture site	Number of individuals			
	Total	2017/2018	2018/2019	2019/2020
Hudiksvall	10	4	9	4
Örebro	30	10	18	22
Nyköping	14	4	8	10
Kristianstad	12	1	8	11
Svedala	10	5	8	4

Note

**FIGURE 1:**
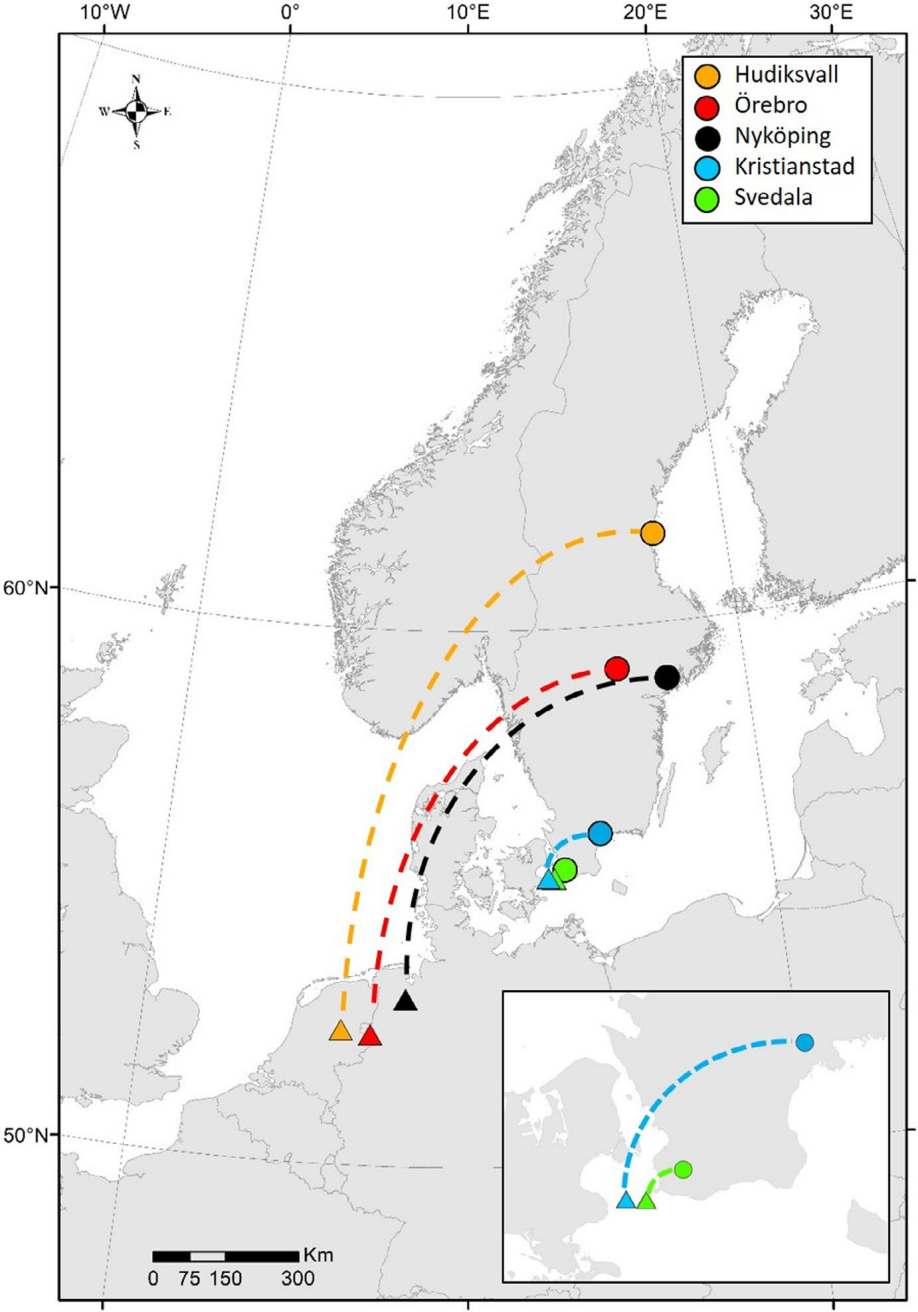
Capture sites (circles) and coordinates (triangles) for mean GPS locations at the predicted mean date when individuals from each capture site have reached the asymptotic migration distance (i.e., farthest distance from the capture sites). For modeling procedure, see Section 2.3. Dashed lines do not represent actual migration routes

See Figure 1 for capture sites.

The five capture sites represent a latitudinal range from central to southern Sweden, embracing the main part of the national breeding range of Greylag geese (55°–61°N, Figure 1, Table 1). The northernmost site is an urban wetland surrounded by grasslands and lawns in a city park in Hudiksvall (N 61°43.96′, E 17°6.55′), located in the Southern boreal zone (Ahti et al., 1968; Hallanaro et al., 2002). This site mainly hosts molting geese, and less than five breeding pairs of Greylag geese. The site at Örebro (N 59°9.59′, E 15°22.86′) is part of a large wetland reserve situated close to the transition zone between the Southern boreal and Boreo‐nemoral zones. The area is dominated by agricultural fields and attracts significant numbers of geese for breeding, as well as for staging in spring and autumn. Kvismare Bird Observatory estimated the minimum numbers of breeding pairs at 240 in 2017–2018. The site at Nyköping (N 58°58.17′, E 17°9.07′) is a wetland surrounded by a fragmented landscape of forests, extensively managed grasslands, agricultural fields, and lakes. This wetland is situated in the Boreo‐nemoral zone and holds 10–20 breeding pairs, and ca. 80–110 molting Greylag geese. The two southernmost sites, at Kristianstad (N 56°4.98′, E 14°21.07′) and Svedala (N 55°33.34′, E 13°14.65′), are situated in the Nemoral zone. Some 20 pairs of Greylag geese breed at each site. The capture site at Kristianstad is in pastures on a narrow strip of land between two lakes, whereas the Svedala site comprises artificial wetlands on a golf course, surrounded by beech forest and agricultural fields.

### 2.2 Data management

For the analyses, we used standardized data of one location per day and individual using the location closest to mid‐day, i.e., 12:00 a.m. UTC (coordinated universal time). In total, 82 individuals from the five capture sites were tracked for at least one annual cycle. We defined the annual cycle as starting on July 1, when all geese were flightless and resident in a restricted area for breeding and molting. We only included individuals with location data for more than 349 days of the annual cycle (i.e., >90% data coverage; mean = 362.1 days of the annual cycle/individual). Locations for all geese were visually inspected in ArcGIS (vers. 10.7), and in cases where individuals obviously moved together in pairs or flocks, the individuals with the least number of locations (*n* = 6) were removed completely from the dataset to avoid inter‐individual autocorrelation in movement. Consequently, data from 76 individuals were used in subsequent analyses (Table 1 and Table S3).

### 2.3 Statistical modeling

We used the modeling framework developed by Bunnefeld et al. (2011) allowing scale‐independent modeling and quantification of five migration variables: (1) migration distance, (2 and 3) timing of autumn and spring migration, and (4 and 5) duration of autumn and spring migration. In addition, the framework allows modeling of the predictability of movement strategies over time and space for flocks and individuals originating at different capture sites. The analysis is based on data on spatiotemporal displacement (i.e., net squared displacement, “NSD”) of individual geese during the annual movement cycle. The NSD value is calculated using the squared distance (km^2^) based on a straight line from the starting location (July 1 each year) to all consecutive daily locations for each individual and annual cycle (July 1 year *t* to June 30 year *t *+ 1). These calculations were made in the R package *adehabitatLT* version 1.6 (Calenge, 2020). Specifically, we used a non‐linear mixed model (package *nlme*) based on the following equation (derived from Bunnefeld et al., 2011):

$$\text{NSD}=\frac{\delta_{a}}{1+\exp\left(\frac{\theta_{a}‐t}{\varphi_{a}}\right)}+\frac{\delta_{s}}{1+\exp\left(\frac{\theta_{s}‐t}{\varphi_{s}}\right)}$$

The terms *δ_a_* and *δ_s_* represent the asymptotic height of the annual movement cycle (i.e., movement distance during autumn and spring), *θ*_a_ the date of reaching half of the asymptotic distance during autumn migration, *θ*_s_ the date of reaching half of the spring migration distance to the sites of origin (i.e., timing), and *φ*_a_ and *φ*_s_ the number of days lapsed to cover a quarter of the distance moved (from 1/2 to 3/4 of the asymptotic migration distance) in autumn and spring (i.e., duration), respectively. The equation is divided into two sub‐equations to allow for variation in movements in autumn versus spring (e.g., differences in timing and duration due to potential alteration of staging sites or migration triggers) (Bunnefeld et al., 2011).

To identify differences in migration strategy between geese of different origin, we used the NSD values for the full annual cycles as response variable, and explored the effect of capture site (five‐level factor: Hudiksvall, Örebro, Nyköping, Kristianstad, Svedala) with the complement of annual cycle (three‐level factor: 2017/2018, 2018/2019, 2019/2020) as explanatory variable for distance, duration, and timing of autumn and spring movement (see explored model structures in Table S1). To avoid pseudo‐replication due to using data from individual geese from more than one annual cycle, we also explored the most parsimonious random‐effect structure of goose ID on the defined migration variables. To define starting values for each respective variable in the model using the equation above, we plotted NSD data over the annual cycle (days lapsed from 1 July) and manually fitted a curve for each capture site to derive prior values to parameterize the equation above. Model exploration showed that only model 1 was supported by the data and converged accordingly (Table S1). In model 1, NSD values (km^2^) were used as response variable, capture site as a fixed‐effect variable of distance, duration and timing of autumn and spring movement, and goose ID as a random effect on migration distance.

To test if Swedish Greylag geese show a leapfrog migration pattern, we used model predictions to identify the month when geese from each capture site reached their asymptotic distance and calculated mean coordinates for geese from each capture site at that time. To obtain mean coordinates, we first calculated the mean value for each individual having data from multiple annual cycles and then estimated the grand mean for all individuals from each capture site. Maps were created in ArcMap (version 10.7).

To test for individual consistency in movement strategy over consecutive annual cycles, we extracted data from individuals that had NSD covering at least two consecutive annual cycles (*n* = 43, Table S2) and estimated the Pearson correlation between the NSD values for individuals at each given point in time (days since July 1) during an annual cycle and the following (i.e., 2017/2018 vs. 2018/2019 and 2018/2019 vs. 2019/2020). All statistical modeling was carried out in R (version 3.6.3; R Core Team, 2020).

## 3 RESULTS

**FIGURE 2:**
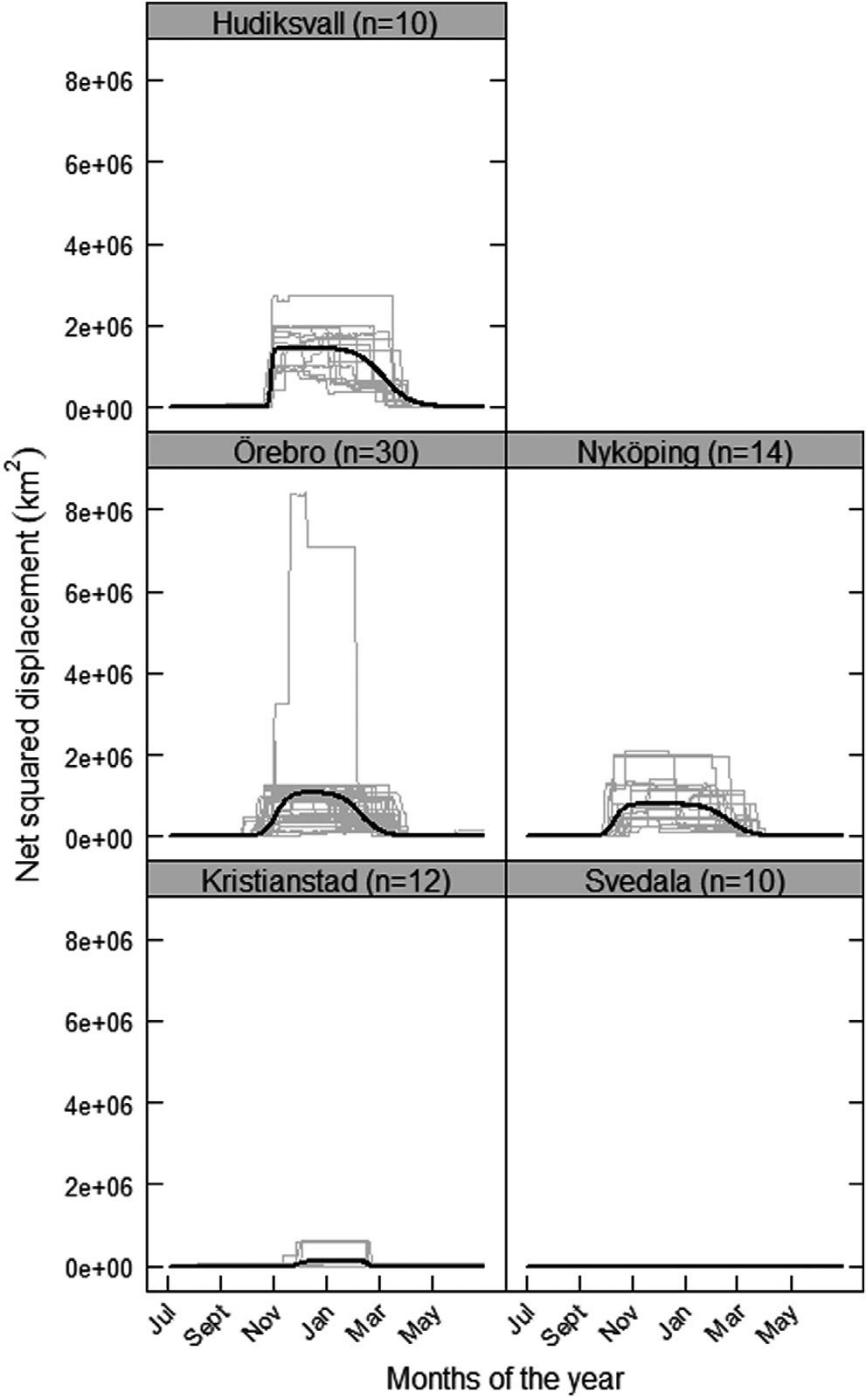
Net squared displacement based on GPS data over the annual cycles (July 1 to June 30, 2017–2020) for individual Greylag geese (grey lines) originating at five capture sites in Sweden; Hudiksvall (*n *= 10), Nyköping (*n *= 14), Örebro (*n *= 30), Kristianstad (*n *= 12), and Svedala (*n *= 10). Model predictions (black lines) show the mean movement strategy for geese from each capture site and are based on a non‐linear mixed model with the net squared displacement distance (km^2^) as response variable, capture site as fixed effect variable on distance, duration and timing of autumn and spring movement, and goose ID as random effects on the asymptotic migration distance. The *Y*‐axis is kept constant for comparison; for detailed graphs with adjusted *Y*‐axes for each capture site, see Figures S1–S5

We found that variation in all migration variables, i.e., distance (km), timing of autumn and spring movement (dates), and duration of autumn and spring movement (days), was explained by the latitudinal origin of geese (i.e., capture site). Our results demonstrate a positive correlation between migration distance and latitudinal origin of geese. Geese from the northernmost site Hudiksvall (1207 km, CI: 985–1395) on average migrated 1.4 and 1.2 times as far as those from the more centrally located sites Nyköping (892 km, CI: *NA*–1388) and Örebro (1041 km, CI: 177–1462), and 15 and 4 times as far as those from the southernmost sites Svedala (82 km, CI: *NA–*1106) and Kristianstad (327 km, CI: *NA*–1121) (Figure 2, Table 2). Consequently, Greylag geese from northern capture sites migrated farther south than did the geese of more southern origin (Svedala and Kristianstad) (Figure 1).

**TABLE 2:** Predicted model estimates (95% confidence intervals) based on GPS data of asymptotic migration distance (km), the start and end dates of the annual cycles (July 1 to June 30, 2017–2020) for individual Greylag geese originating at five capture sites in Sweden; Hudiksvall (*n* = 10), Örebro (*n* = 30), Nyköping (*n* = 14), Kristianstad (*n* = 12), and Svedala (*n* = 10). Predictions were derived from a non‐linear mixed model with the net squared displacement distance (km^2^) as response variable, capture site as fixed effect variable, and goose ID as random effect on the asymptotic migration distance

Migration variable	Hudiksvall	Örebro	Nyköping	Kristianstad	Svedala
Net squared distance (km^2^)	1,457,388 (968,314–1,946,461)	1,085,376 (31,535–2,139,216)	795,883 (−333,536–1,925,302)	107,204 (−1,044,075–1,258,483)	6756 (−1,210,934–1,224,446)
Distance (km)	1207 (984–1395)	1041 (177–1462)	892 (^a^−1388)	327 (^a^−1121)	82 (^a^−1106)
Timing (days since July 1)					
Autumn	119 (119–120)	125 (125–126)	102 (101–103)	150 (148–152)	32 (0–1441)
Spring	250 (249–251)	224 (223–226)	234 (232–236)	232 (230–234)	154 (−10,226–10,534)
Timing (date)					
Autumn	28 Oct (28–29)	3 Oct (3–4)	10 Oct (9–10)	28 Nov (26–30)	1 Aug (1 Jul–^a^)
Spring	7 Mar (6–8)	10 Feb (9–12)	20 Feb (18–22)	18 Feb (16–20)	2 Dec (^a^)
Duration (days)					
Autumn	1.3 (1.1–1.4)	7.1 (6.7–7.6)	5.6 (4.9–6.3)	2.8 (1.2–4.4)	34.4 (−806–875)
Spring	15.5 (14.8–16.1)	12.0 (10.6–12.1)	13.1 (11.5–13.5)	1.3 (−1–1.8)	176.8 (−2364–2716)

Note

^a^ It is not applicable to take the square root of the lower confidence interval of the net squared distance (km^2^) for Nyköping (−333,536 km^2^), Kristianstad (−1,044,075 km^2^), and Svedala (−1,210,934 km^2^). Confidence intervals for timing was out of bound to transform into dates for Svedala (upper confidence interval for autumn: 1442 days after Jul 1, and confidence intervals for spring: −10,226 to 10,534 days after Jul 1).

Movement strategies of individual geese varied considerably both within and among capture sites, with particularly wide confidence intervals around the predicted migration distance (Figure 2, Table 2, for detailed graphs for each capture site, see Figures S1–S5). Geese from Svedala made only restricted, local movements (82 km, CI: *NA*–1106) and did not generate any distinct migration patterns (Figure 2 and Figure S5). As a result, there were wide confidence intervals around the predicted estimates of timing and duration of both autumn and spring movement, covering the full annual cycle of geese from Svedala (i.e., a poor fit of the non‐linear mixed model; Figure 2, Table 3). For the second‐most southern capture site, Kristianstad, our results show that many geese were relatively resident all year around, like the geese originating at Svedala, whereas a few individuals displayed a more pronounced migration pattern (i.e., a net squared displacement curve, Figure 2) resulting in higher predicted migration distance in Kristianstad compared to Svedala (Table 3).

**TABLE 3:** Pearson correlation estimates (mean, min‐max) of individual movement strategies of Greylag geese (i.e., net squared displacement value on each given day) between consecutive annual cycles (2017/2018 to 2018/2019 and 2018/2019 to 2019/2020). The correlation estimates only include individual geese with data from more than one annual cycle (*n* = 43)

Capture site	2017/2018 to 2018/2019		2018/2019 to 2019/2020	
	Mean (min–max)	*n*	Mean (min–max)	*n*
Hudiksvall	0.86 (0.84–0.90)	3	0.86 (0.79–0.95)	4
Örebro	0.89 (0.84–0.92)	5	0.85 (0.68–0.96)	15
Nyköping	0.93 (0.92–0.93)	2	0.81 (0.63–0.95)	8
Kristianstad	−0.84	1	0.38 (−0.10–0.83)	6
Svedala	0.24 (−0.17–0.84)	3	0.31 (−0.01–0.95)	4

We found apparent patterns of timing of both autumn and spring migration in the individual geese that displayed a distinct migration pattern (i.e. a net squared displacement curve) for Hudiksvall, Nyköping, and Örebro, and for migrating individuals from Kristianstad. Geese from Örebro reached half their migration distance by the beginning of October (Oct 3, CI: 3–4), followed by those from Nyköping a week later (10 Oct, CI: 9–10), Hudiksvall by the end of October (Oct 28, CI: 28–29), and the few geese migrating from Kristianstad reaching half their migration distance by the end of November (Nov 28, CI: 26–30). Spring migration timing corresponded to the approximate order of autumn migration timing, i.e., geese had covered half of their migration distance back to Örebro by the second week of February (Feb 10, CI: 9–12), followed by geese from Kristianstad (Feb 18, CI:16–20) and Nyköping (Feb 20, CI: 18–22) roughly a week later, and geese from Hudiksvall by early March (Mar 7, CI: 6–8).

The general consistency in order of timing of autumn and spring migration for geese with different latitudinal origin indicates that the duration of the migration period is relatively similar in geese from all capture sites, except for those from Svedala and Kristianstad, which moved only locally. The geese from Hudiksvall, which migrated the farthest and fastest in autumn (1.3 days, CI: 1.1–1.4), were the ones with the longest migration duration (i.e., time to cover a quarter of the distance) in spring (15.5 days, CI: 14.8–16.1), followed by geese from Nyköping (13.1 days, CI: 11.5–13.5), and Örebro (12.0 days, CI: 10.6–12.1) (Figure 2, Table 2). The migration duration was consistently less in autumn than in spring for geese from Hudiksvall, Örebro, and Nyköping. For geese from Kristianstad, autumn migration duration was slightly longer (2.8 days, CI: 1.2–4.4) than in spring (1.3 days, CI: −1–1.8) (Figure 3, Table 2).

The individual consistency in movement strategy (i.e., the correlation in net squared displacement on a given day) from one annual cycle to the next was high in the geese migrating the farthest, i.e., the northernmost and central capture sites (Hudiksvall, Örebro, and Nyköping; correlation range: 0.63–0.96, Table 3). There were not any indications of differences depending on the specific annual cycles considered in geese from these sites (2017/2018 *vs*. 2018/2019 and 2018/2019 *vs*. 2019/2020). Resident or short‐moving geese from the southern sites (Kristianstad and Svedala) generally showed lower or no individual consistency, with only a few individuals showing consistent movements from one annual cycle to the next (correlation range: −0.84–0.95, for details see Table 3).

## 4 DISCUSSION

We found that migration distance increased with increasing latitude of origin in Swedish Greylag geese, and that the final wintering areas were generally located farther south for the geese migrating from the northern capture sites. Moreover, the migration strategies of geese from different capture sites differed in timing and in duration of autumn and spring movement, but with considerable individual variation within capture sites. Geese from the three northern capture sites (Hudiksvall, Nyköping, and Örebro) showed distinct migration patterns, whereas only a few individuals from the southern site Kristianstad migrated, albeit short distances, and the geese from Svedala were relatively resident, only displaying local movements. Taken together, these findings corroborate the hypothesis that present‐day Swedish Greylag geese exhibit a leapfrog migration pattern *sensu* Salomonsen (1955) and Berthold (2001) and refute the alternative hypothesis of a retained chain migration pattern.

### 4.1 Autumn migration was faster than spring migration in geese from the northernmost sites

Among the three northern capture sites, where all geese clearly migrated, the duration of autumn migration was rather similar despite differences in migration distance. The geese that migrated the farthest (Hudiksvall) thus also migrated the fastest. Yet, they did not start autumn migration earlier, as geese from Örebro and Nyköping had reached a quarter of their migration distance earlier than those from Hudiksvall. This pattern fits with findings from Greylag geese in Norway, where northern breeders migrated later than southern breeders. However, contrary to our results, northern breeders in Norway migrated slower than southern breeders (Andersson et al., 2001).

Interestingly, the time used to cover a quarter of the migration distance was consistently less in autumn than in spring for geese from Hudiksvall, Örebro, and Nyköping. This is in line with GPS location data from Greater white‐fronted geese (*Anser albifrons*) (Kölzsch et al., 2014), but contrary to phenological patterns in many other waterfowl, in which spring migration is faster than autumn migration (Calenge et al., 2010; Nilsson et al., 2013). Drent et al. (1978) argued that herbivorous waterfowl time their spring migration to coincide with the flush of nutritious plant growth as spring progresses northwards, the so called “green wave hypothesis.” Kölzsch et al. (2014) suggested that the slower spring migration in greater white‐fronted geese was a response to gradual northwards greening in spring, thus consistent with the “green wave hypothesis.” For any migration strategy, the high present‐day all‐winter abundance of autumn‐sown winter green agricultural crops in the migration corridor of Greylag geese is a likely factor to counteract adaptive responses to phenology of natural vegetation, at least for most of the northward migration.

### 4.2 Non‐migratory behavior is common in geese from the southernmost sites

Unlike geese from the three northern capture sites, most of those from the southern (Svedala and Kristianstad) were resident in a very restricted area, with only short‐distance excursions locally or regionally. The number of Greylag geese wintering in southern Sweden has increased steadily from almost zero to ⁓60,000 during the last 30 years (Nilsson et al., 2020), but the origin of these birds has been largely unknown. The present study demonstrates that non‐migratory behavior is common among Greylag geese breeding in southernmost Sweden, and that they do very limited movements overall during the annual cycle. Interestingly, we see a slight difference between the two southernmost capture sites; while all geese from Svedala were relatively resident, those from Kristianstad were either residents or short‐distance migrants. However, Greylag geese from Kristianstad most probably do not fit the classical definition of “partial migrants”; it is not known whether individuals showing different strategies (i.e., those that only move locally and those that fly to nearby Denmark) differ with respect to age, body condition, or “genetic programming.”

### 4.3 Individual consistency in movement strategy between years

Individual consistency in movement strategy from one annual cycle to the next was high in the distinctly migratory geese, i.e., those from Hudiksvall, Örebro, and Nyköping, whereas the short‐moving geese with at least two annual cycles from the southern sites (Kristianstad and Svedala) indicated low or no individual consistency (Table 3). Our second hypothesis that individuals should show a consistency in movement habits between years was thus corroborated by the migratory geese from the three northern sites, whereas those from Kristianstad and Svedala in the south provided equivocal results or even refuted the predictions derived from this hypothesis. We interpret these results cautiously, however, as we only tested short‐term individual consistency (i.e., from one annual cycle to the next) and the sample size in terms of both annual cycles (e.g., limited climatic variation) and individuals per capture site was limited.

### 4.4 Leapfrog migration pattern and inter‐individual variation

This study demonstrates a present‐day leapfrog migration pattern in Swedish Greylag geese, and differences in migration strategy at the level of capture site (latitude of breeding or molting area). We argue this pattern is robust because: (a) it conforms to the spatial predictions of the leapfrog hypothesis; (b) at the level of capture site, migration distance increased with latitude; (c) only 2 of 22 geese from the two southern sites reached the winter areas of geese from the three capture sites farther north; and (d) only one bird from the three northern capture sites wintered in the core winter area of geese from the two southern sites.

At the same time, our data highlight notable within‐site variation in timing, duration, and migration distance, as witnessed by the individual NSD curves (the grey lines in Figures S1–S5). In the following, we will argue that the large‐scale leapfrog pattern and the within‐site individual variability found need to be seen as complementary with respect to adaptation and the evolution of a new migration pattern. Consequently, we advocate against seeing the present spatial (leapfrog) pattern as a set of stereotypes by capture site, and instead wish to point out the variation within capture sites as an evolutionary “toolbox.” Accordingly, individual variation in movement represents a multitude of sub‐strategies open for evolutionary selection and different fitness outcomes; in other words a likely driver of long‐term, adaptive, change at the level of breeding area. Work on Whooping cranes (*Grus americana*) highlights the importance of such variability by showing that older and innovative individuals attempting new strategies can be drivers of rapid change in migration patterns (Teitelbaum et al., 2016). Juvenile Greylag geese follow their parents during their first autumn and winter, making the early migration habits a socially learned trait (*c*.*f*., Kölzsch et al., 2020). Sharing of information within flocks speed up adaptations in migration performance as well as feeding and site choice (Delgado et al., 2018; Mueller et al., 2013).

We acknowledge the fact that the previous chain migration pattern of Swedish Greylag geese was not documented by individual GPS location data, but rather by recoveries of geese with tarsus rings (n=80 individuals; Fransson & Pettersson, 2001). Nevertheless, our results imply that there has been an overall change in migration patterns. The historically most important winter site in Coto Doñana in Spain is nowadays rarely used by Swedish Greylag geese, and similar changes have occurred in other parts of the continental European flyway (Voslamber et al., 2010).

### 4.5 Evolutionary perspectives

Greylag geese breeding in southernmost Sweden have gone from being long‐distance migrants to being mainly non‐migrants in less than 35 years, which is equivalent to 8–10 generations. This begs the question why geese in different parts of Sweden, within the same flyway, have responded so differently. To individual birds it is an asset to know an area well, e.g., where the best roost and foraging sites are, and where predation risk is low. Such advantages are especially important in lean times of nutritional bottlenecks and challenging weather, and favor resident individuals compared to migratory conspecifics that come to the area (Alerstam & Enckell, 1979). A side effect of residency (“prior occupancy” in many verbal models) is the lack of migration cost, and selection will favor residency if it confers higher fitness than adopting a migratory behavior. This scenario fits well with our result that Greylag geese in southernmost Sweden have become mostly resident. It also helps explain why conspecific migrants from the north, with little or no local knowledge, do not stop to winter in southern Sweden. The recent adoption of a resident strategy has likely conferred strong selective advantages in Greylag geese breeding in southernmost Sweden, due to mild winters reducing nutritional needs. In addition, the acreage of autumn‐sown winter green crops has increased during the last 35 years, providing abundant predictable high‐quality food the year around. In other words, there has not been any fitness penalty in pursuing a resident strategy.

In a theory based on the ideal despotic distribution, i.e., assuming that prior occupancy is an asset, Holmgren and Lundberg (1993) predicted that a leapfrog migration pattern is most likely to evolve when migration costs are high. Compared to the situation 35 years ago, we do not see that migration costs have remained high in terms of energy expenditure. On the contrary, Greylag geese from our three northerly sites have reduced their migration distance significantly, which should lead to lowered energetic costs. Lundberg and Alerstam (1986) presented the most explicit treatment of factors that may induce transition from chain to leapfrog migration pattern. Their theory, too, is based on migration being costly and on an advantage of priority (residents *vs*. migrants), leading to asymmetrical competition. Under these premises, a chain migration pattern will change into a leapfrog pattern if there is an accentuated increase in breeding suitability toward the north or in wintering suitability toward the south in the flyway (Lundberg & Alerstam, 1986). In the absence of data on possible changes in breeding suitability, per capita mortality, and a comprehensive measure of migration cost, it is, however, hard to say whether the theories offered by Lundberg and Alerstam (1986) and Holmgren and Lundberg (1993) explain the change from chain to leapfrog migration in Swedish Greylag geese. While the selective value of adopting residency in southernmost Sweden under recent and present condition is relatively obvious, it is harder to explain why geese breeding in central and northern Sweden continue to overshoot their southern conspecifics.

The three theories above, and for that matter most hypotheses about evolution of bird migration, are framed within the paradigm of resource limitation and competition (intraspecific and/or interspecific), assuming that density‐dependent processes operate (Alerstam et al., 2003; Salomonsen, 1955). Assumptions and constraints within this paradigm are relaxed if populations are well below their carrying capacity, which may have been the case for some time for Greylag geese in Sweden and beyond. A combination of sustained population growth and improved body condition, the latter implied by lower age at first reproduction (Nilsson & Kampe‐Persson, 2018b), points in this direction. Consequently, if food has long been superabundant, and the per capita hunting mortality rate has fallen as the population has grown, a more successful hypothesis to explain the changed migration pattern may need to be formulated within a paradigm that does not assume food resource limitation.

Although the arguments above are partly speculative, there is evidence that the environment in which European Greylag geese live has seen a reduced seasonality in food abundance, the latter in itself proposed as a key driver for a migratory strategy to evolve (Alerstam & Högstedt, 1982). Swedish Greylag geese nowadays find most of their food most of the year on cropland, instead of in natural habitats. There are indeed empirical reasons to question the omnipresence of resource limitation and density‐dependent processes in geese, as in many other taxa (e.g., Rodhe, 2005). In a review of 54 studies explicitly testing for the occurrence of density‐dependent patterns and processes in ducks, Gunnarsson et al. (2013) found that no less than 70 of 154 species‐specific cases lacked evidence for density dependence. It can be argued that goose populations might be limited by other factors, e.g., availability of nest and molting sites, but no such scientific evidence is apparent for Greylag geese breeding in Sweden.

## 5 CONCLUSIONS

The present study demonstrates significant variation in both movement pattern among individuals from a given capture site, and among capture sites. Greylag geese in W Europe may have lived largely unrestricted from food resource limitation and associated competition during the last decades, permitting rapid adoption of new migration strategies tuned to super‐abundant food in the agricultural landscape and benign winters with low mortality. The change from a classic chain migration to the present leapfrog pattern has taken some 35 years. This time span and the number of generations involved might be too few to allow classical Darwinian between‐generation adaptation to drive the change, even under assumptions of assortative mating. Consequently, and given that young geese follow their parents during their first migration, we presume an important role of within‐family inter‐generation change in migration habits as a driver behind the rapid change in migration patterns in Swedish Greylag geese.

Our study highlights the general value of collecting individual movement data over entire annual cycles from a variety of breeding sites within a population or flyway. It also emphasizes the need to think outside the paradigm of resource limitation, for population trajectories in ecological time as well as for adaptation in evolutionary time, to understand changed movement patterns in species that spend most of the annual cycle in anthropogenic habitats offering super‐abundant food.

## CONFLICT OF INTEREST

None of the authors have any conflicts of interests to declare.

## AUTHOR CONTRIBUTIONS

**Lovisa Nilsson:** Conceptualization (equal); Data curation (lead); Formal analysis (lead); Visualization (lead); Writing – original draft (equal); Writing – review & editing (equal). **Camilla Olsson:** Conceptualization (equal); Data curation (equal); Formal analysis (supporting); Writing – review & editing (equal). **Johan Elmberg:** Conceptualization (equal); Data curation (supporting); Funding acquisition (lead); Project administration (lead); Writing – original draft (equal); Writing – review & editing (equal). **Nils Bunnefeld:** Conceptualization (equal); Methodology (lead); Writing – review & editing (equal). **Niklas Liljebäck:** Conceptualization (equal); Funding acquisition (supporting); Project administration (supporting); Writing – review & editing (equal). **Johan Månsson:** Conceptualization (equal); Data curation (supporting); Funding acquisition (lead); Project administration (lead); Writing – review & editing (equal).

## Supporting information

Supplementary Material

## Data Availability

Data is available in Dryad: https://doi.org/10.5061/dryad.wh70rxwq3.
