# Supplementary material for: Leapfrog migration and residents: New migratory habits in Swedish Greylag geese
Source: Ecol Evol. 2022 Mar 23;12(3):e8740. doi: 10.1002/ece3.8740 (PMC8941501; doi:10.1002/ece3.8740)
Supplement: Supplementary file 1 — Supplementary Material [file ECE3-12-e8740-s001.docx]

**Supporting Information**

*Leapfrog migration and residents: new migratory habits in Swedish Greylag geese*

Lovisa Nilsson^1^, Camilla Olsson^1,2^, Johan Elmberg^2^, Nils Bunnefeld^3^, Niklas Liljebäck^1^ & Johan Månsson^1^

^1^ Grimsö Wildlife Research Station, Swedish University of Agricultural Sciences, SE-73993, Riddarhyttan, Sweden

^2^ Department of Environmental Science and Bioscience, Kristianstad University, SE-29188 Kristianstad, Sweden

^3^ Biological and Environmental Sciences, University of Stirling, Stirling, FK9 4LA, United Kingdom

| Model | Fixed effect | Random effect | Convergence |
| --- | --- | --- | --- |
| 1 | Capture site on distance, timing, duration | Goose ID on distance | Yes |
| 2 | Capture site on distance, timing, duration | Goose ID on distance, timing | No |
| 3 | Capture site on distance timing, duration | Goose ID on distance, timing, duration | No |
| 4 | Capture site and annual cycle on distance, timing, duration | Goose ID on distance | No |
| 5 | Capture site on distance, timing, duration | Goose ID and annual cycle on distance | No |

Table S1. Structure of the non-linear mixed models tested for convergence (see equation in Methods) based on different combinations of the fixed factors capture site (five-level factor: Hudiksvall, Örebro, Nyköping, Kristianstad, Svedala) and annual cycle (three-level factor: 2017/2018, 2018/2019, 2019/2020), and the random effect goose ID (76 levels) on the movement variables: distance, timing and duration of autumn and spring movement.

Table S2. Individual geese with GPS location data covering two or three annual cycles, per capture site.

| Capture site | Number of individuals | |
| --- | --- | --- |
|  | *2 cycles* | *3 cycles* |
| Hudiksvall | 7 | 0 |
| Örebro | 14 | 3 |
| Nyköping | 6 | 1 |
| Kristianstad | 5 | 1 |
| Svedala | 3 | 2 |

Table S3. Individual information about Greylag goose individuals (n=76) included in the statistical modelling in the present study.

| Catch site | Sex | Age at tagging | Annual cycles | Individual |  |
| --- | --- | --- | --- | --- | --- |
| Hudiksvall | Female | 2 cy+ | 2 | D13 |  |
| Hudiksvall | Female | 2 cy+ | 2 | D25 |  |
| Hudiksvall | Female | 2 cy+ | 1 | D67 |  |
| Hudiksvall | Female | 2 cy+ | 2 | D77 |  |
| Hudiksvall | Female | 2 cy+ | 2 | D78 |  |
| Hudiksvall | Female | 2 cy+ | 2 | D79 |  |
| Hudiksvall | Female | 2 cy+ | 2 | DM3 |  |
| Hudiksvall | Female | 2 cy+ | 1 | DM4 |  |
| Hudiksvall | Female | 2 cy+ | 1 | DM5 |  |
| Hudiksvall | Female | 2 cy+ | 2 | DM6 |  |
| Örebro | Female | 2 cy+ | 2 | K03 |  |
| Örebro | Female | 2 cy+ | 2 | K06 |  |
| Örebro | Female | 2 cy+ | 2 | K09 |  |
| Örebro | Female | 2 cy+ | 1 | K35 |  |
| Örebro | Female | 2 cy+ | 1 | K36 |  |
| Örebro | Female | 2 cy+ | 1 | K37 |  |
| Örebro | Female | 1 cy | 3 | S34 |  |
| Örebro | Female | 2 cy+ | 3 | S57 |  |
| Örebro | Female | 2 cy+ | 1 | S65 |  |
| Örebro | Female | 2 cy+ | 3 | S66 |  |
| Örebro | Female | 2 cy+ | 1 | S89 |  |
| Örebro | Female | 2 cy+ | 2 | S96 |  |
| Örebro | Male | 2 cy+ | 2 | K04 |  |
| Örebro | Male | 2 cy+ | 2 | K05 |  |
| Örebro | Male | 2 cy+ | 1 | K08 |  |
| Örebro | Male | 2 cy+ | 1 | K31 |  |
| Örebro | Male | 2 cy+ | 1 | K32 |  |
| Örebro | Male | 2 cy+ | 1 | K34 |  |
| Örebro | Male | 1 cy | 2 | S38 |  |
| Örebro | Male | 1 cy | 1 | S41 |  |
| Örebro | Male | 1 cy | 1 | S51 |  |
| Örebro | Male | 2 cy+ | 1 | S67 |  |
| Örebro | Male | 2 cy+ | 1 | S68 |  |
| Örebro | Male | 2 cy+ | 2 | S90 |  |
| Örebro | Male | 2 cy+ | 2 | S91 |  |
| Örebro | Male | 2 cy+ | 2 | S92 |  |
| Örebro | Male | 2 cy+ | 2 | S93 |  |
| Örebro | Male | 2 cy+ | 2 | S94 |  |
| Örebro | Male | 2 cy+ | 2 | S99 |  |
| Örebro | NA | 2 cy+ | 2 | S64 |  |
| Nyköping | Female | 2 cy+ | 3 | D70 |  |
| Nyköping | Female | 2 cy+ | 2 | D71 |  |
| Nyköping | Female | 2 cy+ | 1 | D74 |  |
| Nyköping | Female | 2 cy+ | 1 | K20 |  |
| Nyköping | Female | 2 cy+ | 1 | K21 |  |
| Nyköping | Female | 2 cy+ | 1 | K22 |  |
| Nyköping | Female | 2 cy+ | 1 | K23 |  |
| Nyköping | Female | 2 cy+ | 1 | S30 |  |
| Nyköping | Female | 2 cy+ | 1 | S32 |  |
| Nyköping | Female | 2 cy+ | 2 | S33 |  |
| Nyköping | Female | 2 cy+ | 2 | S71 |  |
| Nyköping | Female | 2 cy+ | 2 | S72 |  |
| Nyköping | Female | 2 cy+ | 2 | S74 |  |
| Nyköping | Female | 2 cy+ | 2 | S75 |  |
| Kristianstad | Female | 2 cy+ | 1 | K43 |  |
| Kristianstad | Female | 2 cy+ | 1 | K44 |  |
| Kristianstad | Female | 2 cy+ | 1 | S78 |  |
| Kristianstad | Female | 2 cy+ | 2 | S80 |  |
| Kristianstad | Female | 2 cy+ | 2 | S85 |  |
| Kristianstad | Female | 2 cy+ | 1 | S87 |  |
| Kristianstad | Male | 1 cy | 1 | S84 |  |
| Kristianstad | Male | 2 cy+ | 1 | K42 |  |
| Kristianstad | Male | 2 cy+ | 3 | S02 |  |
| Kristianstad | Male | 2 cy+ | 2 | S77 |  |
| Kristianstad | Male | 2 cy+ | 2 | S81 |  |
| Kristianstad | Male | 2 cy+ | 2 | S83 |  |
| Svedala | Female | 2 cy+ | 1 | K14 |  |
| Svedala | Female | 2 cy+ | 1 | S06 |  |
| Svedala | Female | 2 cy+ | 3 | S07 |  |
| Svedala | Female | 2 cy+ | 3 | S14 |  |
| Svedala | Female | 2 cy+ | 1 | S20 |  |
| Svedala | Male | 2 cy+ | 2 | K18 |  |
| Svedala | Male | 2 cy+ | 1 | S05 |  |
| Svedala | Male | 2 cy+ | 2 | S25 |  |
| Svedala | Male | 2 cy+ | 1 | S29 |  |
| Svedala | NA | 2 cy+ | 2 | S12 |  |

**
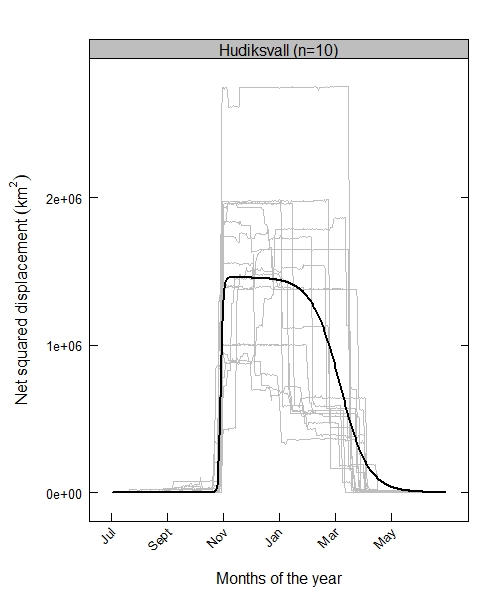
**

Figure S1. Net squared displacement over the annual cycles (1 July - 30 June, 2017-2020) for individual Greylag geese (grey lines) tagged with a GPS collar at Hudiksvall (n=10). The model prediction (black line) demonstrates the mean movement strategy of all geese from this capture site and is based on a non-linear mixed model with the net squared displacement distances (km^2^) as response variable, capture site as fixed effect variable on distance, duration and timing of autumn and spring movement, and goose ID as random effect on the asymptotic migration distance. The y axis is adjusted to fit the specific results for this capture site. For a comparison among capture sites based on a common y axis scale, see Fig. 2.

**
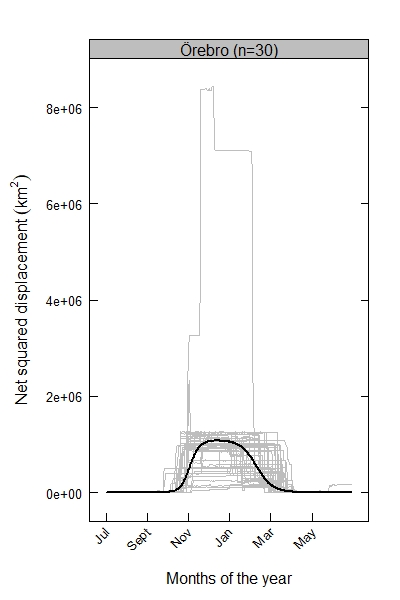
**

Figure S2. Net squared displacement over the annual cycles (1 July - 30 June, 2017-2020) for individual Greylag geese (grey lines) tagged with a GPS collar at Örebro (n=30). The model prediction (black line) demonstrates the mean movement strategy of all geese from this capture site and is based on a non-linear mixed model with the net squared displacement distances (km^2^) as response variable, capture site as fixed effect variable on distance, duration and timing of autumn and spring movement, and goose ID as random effect on the asymptotic migration distance. The y axis is adjusted to fit the specific results for this capture site. For a comparison among capture sites based on a common y axis scale, see Fig. 2.


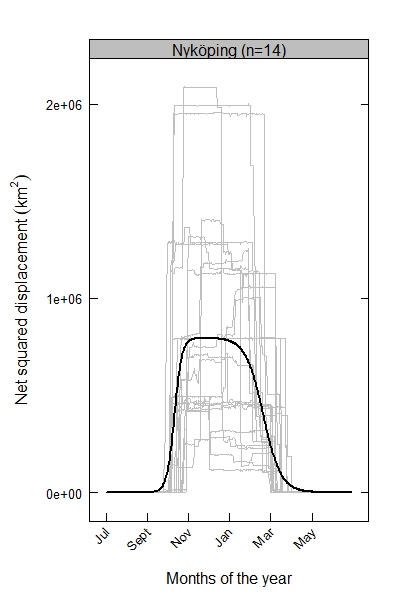


Figure S3. Net squared displacement over the annual cycles (1 July- 30 June, 2017-2020) for individual Greylag geese (grey lines) tagged with a GPS collar at Nyköping (n=14).The model prediction (black line) demonstrates the mean movement strategy of all geese from this capture site and is based on a non-linear mixed model with the net squared displacement distances (km^2^) as response variable, capture site as fixed effect variable on distance, duration and timing of autumn and spring movement, and goose ID as random effect on the asymptotic migration distance. The y axis is adjusted to fit the specific results for this capture site. For a comparison among capture sites based on a common y axis scale, see Fig. 2.


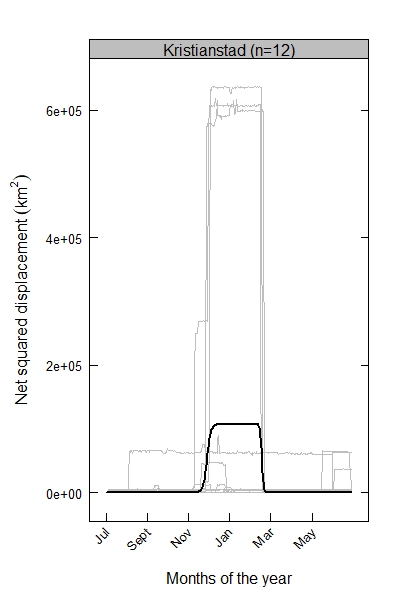


Figure S4. Net squared displacement over the annual cycles (1 July- 30 June, 2017-2020) for individual Greylag geese (grey lines) tagged with a GPS collar at Kristianstad (n=12). The model prediction (black line) demonstrates the mean movement strategy of all geese from this capture site and is based on a non-linear mixed model with the net squared displacement distances (km^2^) as response variable, capture site as fixed effect variable on distance, duration and timing of autumn and spring movement, and goose ID as random effect on the asymptotic migration distance. The y axis is adjusted to fit the specific results for this capture site. For a comparison among capture sites based on a common y axis scale, see Fig. 2.


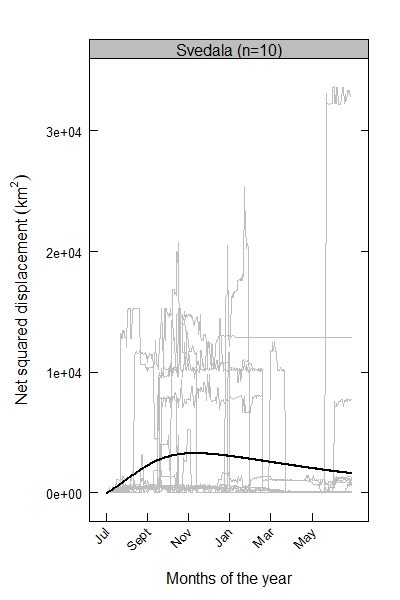


Figure S5. Net squared displacement over the annual cycles (1 July- 30 June, 2017-2020) for individual Greylag geese (grey lines) tagged with a GPS collar at Svedala (n=10). The model prediction (black line) demonstrates the mean movement strategy of all geese from this capture site and is based on a non-linear mixed model with the net squared displacement distances (km^2^) as response variable, capture site as fixed effect variable on distance, duration and timing of autumn and spring movement, and goose ID as random effect on the asymptotic migration distance. The y axis is adjusted to fit the specific results for this capture site. For a comparison among capture sites based on a common y axis scale, see Fig. 2.
